# Comparative genomics of dominant members of the gut core microbiome of the bark beetle, *Dendroctonus rhizophagus* (Curculionidae: Scolytinae) reveals potential functional complementarity in the detoxification process

**DOI:** 10.1186/s12864-025-12279-1

**Published:** 2025-11-19

**Authors:** Karina Vazquez-Ortiz, Flor N. Rivera-Orduña, Gerardo Zúñiga

**Affiliations:** 1 Laboratorio de Ecología Microbiana, Departamento de Microbiología, Escuela Nacional de Ciencias Biológicas, Instituto Politécnico Nacional, Mexico City, Mexico; 2 Laboratorio de Variación Biológica y Evolución, Departamento de Zoología, Escuela Nacional de Ciencias Biológicas, Instituto Politécnico Nacional, Mexico City, Mexico

**Keywords:** *Dendroctonus*, Gut microbiota, Symbionts, Genome, Detoxification, Terpenes, Aromatic compounds

## Abstract

**Background:**

*Dendroctonus rhizophagus* is a bark beetle of great forest importance, due to its aggressive root colonization of seedlings and young pine trees. To successfully complete their life cycle, these insects and associated microorganisms must overcome the trees’ complex defense system. However, little is known about the enzymes and metabolic pathways involved in xenobiotic detoxification by microbial symbionts of bark beetles. We investigated the genomic potential for detoxification in bacteria *Acinetobacter*, *Pseudomonas*, *Rahnella* and *Serratia*, as well as the yeasts *Candida* [*Ogataea* clade], *Danielia*, *Cyberlindnera* and *Zygoascus*, dominant members of the gut core microbiome of *D*. *rhizophagus*, through assembly, annotation, and comparison of their genomes.

**Results:**

Genome analysis identified 1,293 genes in bacteria and yeasts related to 22 KO xenobiotic-degradation pathways. These pathways include those for aromatic compounds (benzoate, ferulate, vanillin), monoterpenes (pinene, camphor, geraniol, limonene) and diterpenes (*dit* genes). Additionally, the genomes contained genes encoding members of the following enzymatic families: cytochrome P450, carboxylesterases, laccases, aldehyde and alcohol dehydrogenases, flavin binding-monooxygenases, intradiol and extradiol dioxygenases, glutathione S-transferases, and multidrug resistance transporters (ABC, MFS, MATE, and RND) implicated in the detoxification process.

**Conclusions:**

Integration of these genes across the phases of xenobiotic detoxification reveals potential functional complementarity between bacteria and yeasts, suggesting that members of the gut microbiota of *D. rhizophagus* may enhance the beetle’s fitness by facilitating the metabolism and tolerance to the tree´s toxic compounds.

**Supplementary Information:**

The online version contains supplementary material available at 10.1186/s12864-025-12279-1.

## Background

*Dendroctonus* bark beetles (Curculionidae: Scolytinae) play a central role in the ecology of coniferous forests (Fam: Pinaceae) in the Northern Hemisphere by participating in nutrient cycling by colonizing and killing weakened or damaged trees [1, 2]. They contribute to ecosystem functioning by promoting heterogeneous age structures and have, by their overall ecological role, positive effects on biodiversity conservation [3, 4]. However, under conditions of forest stress caused by environmental (e.g., drought, high temperatures) or anthropogenic factors, some species act as major disturbance agents, producing outbreaks that kill hundreds of thousands of healthy trees, transform landscapes, alter water, climate, and nutrient cycles, and generate substantial economic losses for forest owners and the forest industry [5, 6].

Except for a brief dispersal period, the life cycle of these bark beetles takes place beneath the bark of trees, where the larvae and adults feed on phloem [7]. The host tree’s defensive chemical system (e.g., the resin flow and the presence of toxic compounds such as terpenes and phenolic and aromatic compounds) can cause severe damage to cell membranes and even the death of insects [8–10]. Therefore, to survive and complete their life cycle, bark beetles must successfully colonize the tree and allow their brood to develop in the subcortical environment.

To facilitate colonization, overcome tree defenses, and obtain sufficient nutrients for brood development, *Dendroctonus* bark beetles rely on a wide set of organisms, mainly pathogenic fungi, bacteria and yeasts [11, 12]. In fact, it has been recognized that these bark beetles and their symbionts are an ecological unit (holobiont) that encompasses a wide range of interspecific interactions [13, 14].

Ecto- and endosymbionts provide biological benefits to *Dendroctonus* bark beetles related to ecological processes, such as the alteration of tree defenses and facilitation of colonization [15, 16], degradation of structural and non-structural carbohydrates [17–19], nitrogen recycling and fixation [18, 20], detoxification of tree xenobiotics [21, 22], enhancement of immunity and protection against pathogens [23–25], and production of pheromones [26–28].

While these benefits have often been derived and generalized from the functional capacities of specific symbionts, the coexistence of microbes in space and time and their interaction with the host, determines the formation of non-static microbiomes promoting genetic, biochemical and physiological changes that influence the life cycle of insects [see [29] and references therein]. In addition, the way of acquiring microbes makes that the diversity structure and composition of microbiome vary among individuals, populations, and species, because deterministic and stochastic ecological processes (e.g., selection, ecological drive, dispersion, climatic variable), and characteristics of the host’s life history (e.g., habitat, distribution range, ontogeny and phylogeny) [30–33].

The acquisition of microbes from *Dendroctonus* species and other bark beetles is environmental [34, 35], although some species transport fungi and bacteria within their gut and mycetangia [15, 24, 36, 37]. Metabarcoding studies have recognized a common (taxonomic) microbiome core of bacteria and fungi associated with the gut of *Dendroctonus* bark beetles, comprising hub and dominant members belonging to the bacterial genera *Enterobacter*, *Pantoea*, *Pseudomonas*, *Rahnella*, *Raoultella* and *Serratia*, and by the fungal genera *Candida*, *Nakazawaea*, *Cladosporium*, *Ogataea* and *Yamadazyma* [34, 38]. The contribution of each to *Dendroctonus* gut´s core bacteriome and mycobiome is slightly different, but apparently consistent across the distribution range and life cycle of species [39–41]. Furthermore, “omics” analysis of some hub members of the gut or complete gut communities has improved knowledge about the potential and real functional role of the microbes of some *Dendroctonus* species [42–45].

In this study, we investigated the potential functional role related to detoxification process of dominant or hub microbes associated with the gut of the bark beetle *D. rhizophagus*. Specifically, we performed comparative genomic analyses of persistent microbiome members across the beetle’s life cycle [39] to infer their individual capacities and functional complementarity during the three detoxification phases: (I) functionalization, (II) conjugation, and (III) excretion.

## Methods

### Isolation and identification of strains

*Dendroctonus rhizophagus* is a species endemic to the Sierra Madre Occidental of Mexico, where it colonizes and kills seedlings of 11 pine species. Its life cycle is univoltine and synchronous, comprising of five larval stages, a pupal stage that gives rise to sexually immature teneral adults, which later mature and emerge from the dead tree to colonize new hosts [46]. Hub and dominant members of the core bacteriome (*Acinetobacter*, *Pseudomonas*, *Rahnella*,* Serratia*) and mycobiome (*Candida* [*Ogataea* clade], *Candida* [= *Danielia*], *Cyberlindnera*, *Zygoascus*) were isolated from the larvae and emerged-adults gut of this bark beetle, which infests *Pinus arizonica* Engelm in the San Juanito locality (27º55’ 54.9’’ N and 107º 35’ 54.6’’ W; 2452 m.a.s.l), Bocoyna Municipality, Chihuahua, Mexico [19].

The strains used in this work were originally identified by sequencing the 16S rRNA gene (bacteria) and ITS region (yeasts). Subsequent genomic analyses confirmed them as *Acinetobacter* sp. nov. ChDrLvgB58 (SAMN48785233), *Pseudomonas carnis* ChDrAdgB60 (SAMN48785438), *Pseudomonas* sp. nov. ChDrLvgB09 (SAMN48785390), *Serratia* sp. nov. CDBB-1961 (SAMN48795659), *Danielia* sp. nov. ChDrAdgY58 (SAMN48796341), *Candida* sp. nov. ChDrAdgY41 (SAMN48796467), and *Zygoascus* sp. nov. ChDrAdgY45 (SAMN48796798). All strains have been deposited in the collection of the Escuela Nacional de Ciencias Biológicas del Instituto Politécnico Nacional (National School of Biological Sciences of the National Polytechnic Institute, Mexico) and are currently being described for publication. In addition, we included genomes from *Rahnella contaminans* ChDrAdgB13 and *Cyberlindnera americana* ChDrAdgY46 previously reported by our working group [44, 47], these genomes were re-assembled (SAMN48786648, SAMN48795594) and re-analyzed for the present study.

### Extraction, sequencing, and assembly of genomes

Genomic DNA (gDNA) from bacterial and yeast strains was extracted from pure cultures using the DNeasy Blood and Tissue Kit (QIAGEN, United States) following the manufacturer protocol. The gDNAs obtained were quantified with a NanoDrop 2000 (Thermofisher Scientific, Wilmington, DE, USA). All genomes were sequenced at Macrogen Inc. (Seoul, South Korea) using the Illumina NovaSeq 6000 platform, generating 2 × 150 paired-end reads. Raw read quality was checked using FastQC v.0.12.1 (http://www.bioinformatics.babraham.ac.uk/projects/fastqc/). Low-quality reads were filtered with Fastp v.0.23.4 software [48], removing adapters, trimming bases < Q20, cut window size = 4, and minimum length = 25 bases. High-quality reads were *de novo* assembled with SPAdes v.4.0 [49], using the *k-mer* values recommended by the software protocol based on read length. The assemblies were compared with several reference genomes deposited in NCBI with OrthoANIu v1.2 [50] and scaffolding with RagTag v.2.1.0 [51], and using the closest reference sequences to our strains (Supplemental Table 1). The quality of the assemblies was assessed by QUAST v.5.2.0 [52].

### Structural annotation of genomes

The genes and protein sequences of bacterial assemblies were predicted with the platform of the Bacterial and Viral Bioinformatics Resource Center (BV-BRC) [53], using the RAST toolkit database. Yeast gene and protein sequences were predicted using GeneMark-ES v.4.72 [54] and AUGUSTUS v.3.5.0 [55]. The completeness of the structural annotation was evaluated with BUSCO v.5.6.1 [56].

### Functional annotation and GO assignments

Functional annotations of predicted proteins were conducted using the eggNOG-mapper v2.1.12 against eggnog v5.0 database [57] (BLOSUM62 matrix and threshold of 1e-5). In addition, the functional annotation and Gene Ontology (GO) from predicted proteins were validated with InterProScan v.5.69–101.0 [58] and BlastP from BLAST + v.2.16.0 [59] against UniprotKB database (https://www.uniprot.org/). GO terms were classified in subsets with GOSlimmer v.1.0.1 (https://github.com/DanFaria/GOEnrichment) using the strategy of full ontology (go-basic.obo) and the GoSlim Set Ribbon for prokaryotes and eukaryotes (goslim_prokaryote_ribbon; goslim_euk_cellular_processes_ribbon.obo).

### Identification of pathways and protein families associated with the detoxification process

Based on the functional annotations generated with eggNOG-mapper, the KEGG Orthology (ko) entries for all bacteria and yeasts genes were described and mapped with the KEGG Mapper-Reconstruct tool [60]. Those ko related to xenobiotics biodegradation and metabolism (09111), limonene degradation (ko00903), and pinene, camphor and geraniol degradation (ko00907) were recovered and quantified.

Coding sequences in each genome were classified to protein families with hmmscan (threshold = 1e-5) implemented in HMMER v3.4, using protein domains of the Pfam database [61]. Genes encoding putative transporter and enzymatic families associated with the detoxification processes such as ATP-binding cassette transporters (ABC, PF00005, PF00664, PF06472, PF00004), Multi-antimicrobial extrusion proteins (MATE, PF01554), Major Facilitator Superfamily (MFS, PF07690, PF12832, PF13347, PF05977, PF06779, PF05631, PF16983, PF00083), Resistance-Nodulation-Division transporters (RND, PF00873), Cytochrome P450 (P450, PF00067), FAD-binding domain enzymes (FAD-binding, PF00667), Aldo/keto reductase family (AKR, PF00248), Carboxylesterase family (COE, PF00135), Flavin-binding monooxygenase (FMO, PF00743), Glutathione S-transferases (GST, PF00043, PF13410, PF14497, PF14834, PF02798, PF13409, PF13417), and Multicopper oxidases (MCO, PF00394, PF07731, PF07732, PF02578) were quantified for each species and plotted in a heatmap using the Seaborn library (https://seaborn.pydata.org/).

### Subclassification of transporters and enzymatic families

Multidrug Resistance (MDR) transporters belonging to the ABC, MATE, MFS, and RND superfamilies were classified into specific families through BlastP analysis against the Transporter Classification database (TCDB; https://www.tcdb.org/) with a threshold of 1e-5.

The classification of FAD-binding, AKR, COE, FMO, GST, and MCO families was performed by obtaining the Enzyme Commission (EC) and protein entries from Eggnog and Interproscan annotations. Sequences lacking a specific subclassification were aligned with the PANTHER HMM scoring tool [62] and Batch CD-Search against the CDD v.3.21 database (https://www.ncbi.nlm.nih.gov/Structure/bwrpsb/bwrpsb.cgi).

To classify the Cytochrome P450, a maximum-likelihood phylogenetic analysis was performed in PhyML v.3.0 [63] with the sequences annotated in UNIPROTKB at the subfamily level. Also, closely related sequences from the different families described by Nelson [64] and Linder [65] were included and aligned with MUSCLE v3.8.31 [66]. The best amino acid substitution model was selected with ModelTest-NG v0.1.7 [67], and tree robustness was assessed with 1,000 bootstrap pseudoreplicates.

### Physicochemical features of protein families

The theoretical isoelectric point (pI) and molecular mass of FAD-binding, AKR, COE, FMO, GST, MCO, P450 and MDR transporters were predicted with the Protein Analysis module of Biopython v.1.81 [68]. In addition, the subcellular localization of these enzymes and transporters was predicted with DeepLocPro v.1.0 for bacterial proteins and DeepLoc v.2.1 for yeast proteins [69].

## Results

### Assembly and structural annotation of genomes

Based on OrthoANI analysis results, the most similar reference genomes to our bacterial and yeast strains, and therefore used for scaffolding, were: *Acinetobacter lwoffii* H7 (GCF_019343495), *Pseudomonas carnis* 20TX0167 (GCF_024722005), *Pseudomonas* sp. M47T1 (GCF_000263855), *Rahnella contaminans* Lac-M11 (GCF_011065485), *Serratia proteamaculans* EBP3064 (GCF_949794035), *Danielia oregonensis* NRRL Y-5850 (GCA_003707785), *Candida piceae* NRRL YB-2107 (GCA_030567815), *Cyberlindnera americana* NRRL Y-2156 (GCA_003708795), and *Zygoascus tannicola* NRRL Y-17392T (GCA_030569095) (Supplemental Table 1).

Genome assemblies of all strains ranged from 11 to 123 scaffolds (> 500 bp), with total length varying from 3.37 to 6.81 Mb in bacteria and between 10.84 and 12.74 Mb in yeasts. The N50 values ranged from 209,644 to 5,277,812, and the annotation had completeness values > 90% for all strains (Table 1). Structural annotations identified 3,427 to 6,276 coding sequences (CDs) in bacteria, with the fewest in *Acinetobacter* sp. ChDrLvgB58 and the most in *Pseudomonas* sp. ChDrLvgB09. Yeast CDs varied from 5,495 to 5,901, the fewest was found in *Danielia* sp. ChDrAdgY58 and the most in *C. americana* ChDrAdgY46 (Table 1).

**Table 1:** Statistical data of genomic assemblies and structural annotations from bacteria and yeasts

Strain	No. of scaffolds (> 500 bp)	Largest scaffold (bp)	Assembly size (bp)	N50	GC content (%)	No. of genes predicted (CDs)	BUSCO complete genes (%)
*Acinetobacter* sp. ChDrLvgB58	68	2,902,178	3,373,974	2,902,178	42.79	3,427	98.7
*P. carnis* ChDrAdgB60	47	804,224	5,868,401	209,644	60.28	5,671	97.7
*Pseudomonas* sp. ChDrLvgB09	56	764,050	6,810,070	258,082	64.86	6,276	99.3
*R. contaminans *ChDrAdgB13	123	1,989,263	5,854,220	1,346,915	52.78	6,007	99.8
*Serratia* sp. CDBB-1961	11	5,277,812	5,310,334	5,277,812	55.26	4,996	99.3
*C. americana *ChDrAdgY46	44	1,619,871	11,428,850	840,453	41.42	5,901	97.1
*Danielia* sp. ChDrAdgY58	81	1,706,783	10,844,627	951,006	45.75	5,495	95.0
*Candida* sp. ChDrAdgY41	101	576,562	12,744,441	289,759	40.53	5,610	94.1
*Zygoascus* sp. ChDrAdgY45	101	1,980,411	11,313,061	814,599	40.17	5,558	93.2

### Functional annotation and GO term classification

For each bacterium and yeast, more than 90% of the predicted CDs were functionally annotated with at least one of the three annotators used. The number of annotated genes ranged from 3,270 to 6,039 in bacteria and from 5,013 to 5,524 in yeast (Supplemental Table 2).

**Fig. 1:**
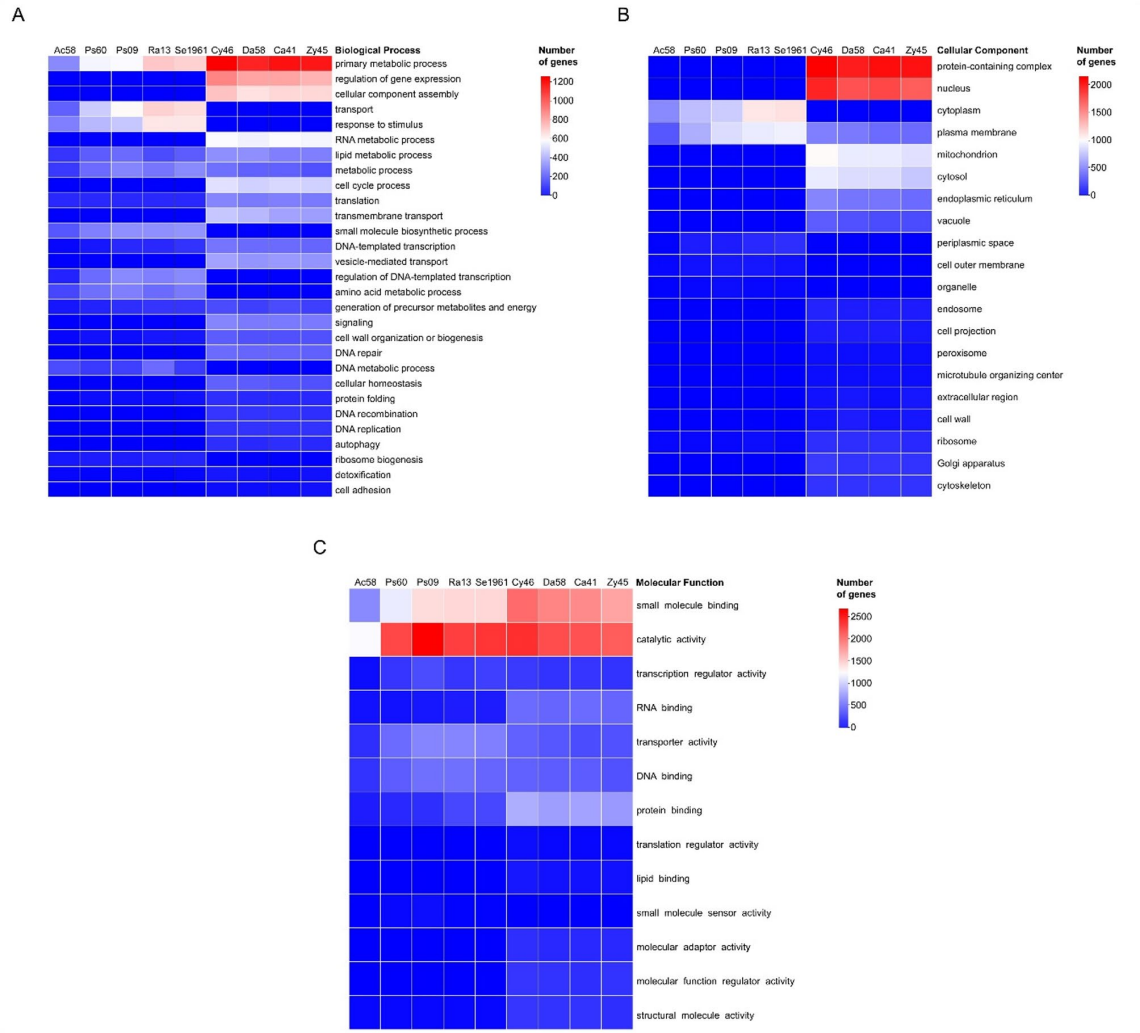
Heatmaps of annotated Gene Ontology (GO) in bacteria and yeasts. GoSlim terms from genes were classified into three main ontologies: (**A**) biological process, (**B**) cellular components, and (**C**) molecular function. Only Go terms with percentage of genes ≥ 1 of each strain were displayed. Ac58 = *Acinetobacter* sp. ChDrLvgB58, Ps60 = *P. carnis* ChDrAdgB60, Ps09 = *Pseudomonas* sp. ChDrLvgB09, Ra13 = *R. contaminans* ChDrAdgB13, Se1961 = *Serratia* sp. CDBB-1961, Cy46 = *C. americana* ChDrAdgY46, Da58 = *Danielia* sp. ChDrAdgY58, Ca41 = *Candida* sp. ChDrAdgY41, Zy45 = *Zygoascus* sp. ChDrAdgY45

The GO slim terms for all microorganisms were classified into three main ontologies: (1) biological process, (2) cellular components, and (3) molecular functions. The number of genes annotated in these GO terms was different among microorganisms. Among the bacteria, *Acinetobacter* sp. ChDrLvgB58 presented the fewest number of genes (2,291) associated with these terms, followed by *P. carnis* ChDrAdgB60 with 4,083 genes, *Pseudomonas* sp. ChDrLvgB09 with 4,817 genes, *R. contaminans* ChDrAdgB13 with 4,217 genes, and *Serratia* sp. CDBB-196 with 4,223 genes. Bacterial genes with relative values ≥ 1% were subclassified into 16 biological processes, seven related to cellular components, and nine to molecular functions (Fig. 1). Within these subclassifications, the most abundant GO terms with a relative number of genes (> 10%) corresponded to primary metabolic process, transport, and response to stimulus within biological process (Fig. 1A); cytoplasm and plasma membrane within cellular components (Fig. 1B); and catalytic activity, small molecule binding, DNA binding, and transporter activity within molecular functions (Fig. 1C).

In yeasts, the fewest number of genes associated with GO terms occurred in *Zygoascus* sp. ChDrAdgY45 with 4,596 genes; followed by *Candida* sp. ChDrAdgY41 with 4,730 genes, *Danielia* sp. ChDrAdgY58 with 4,754 genes, and *C. americana* ChDrAdgY46 with 5,171 genes. Genes with relative abundance ≥ 1% were subclassified into 22 biological processes, 16 related to cellular components and 12 to molecular functions (Fig. 1).

The most abundant GO slim terms in yeasts with a relative number of genes > 10% were primary metabolic process, regulation of gene expression, cellular component assembly, RNA metabolic process, and cell cycle process within biological process (Fig. 1A); protein-containing complex, nucleus, mitochondrion, cytosol, endoplasmic reticulum, and plasma membrane within cellular components (Fig. 1B), and catalytic activity, small molecule binding, protein binding, RNA binding, DNA binding, and transporter activity within molecular functions (Fig. 1C).

### KO pathways associated with xenobiotic degradation

**Fig. 2:**
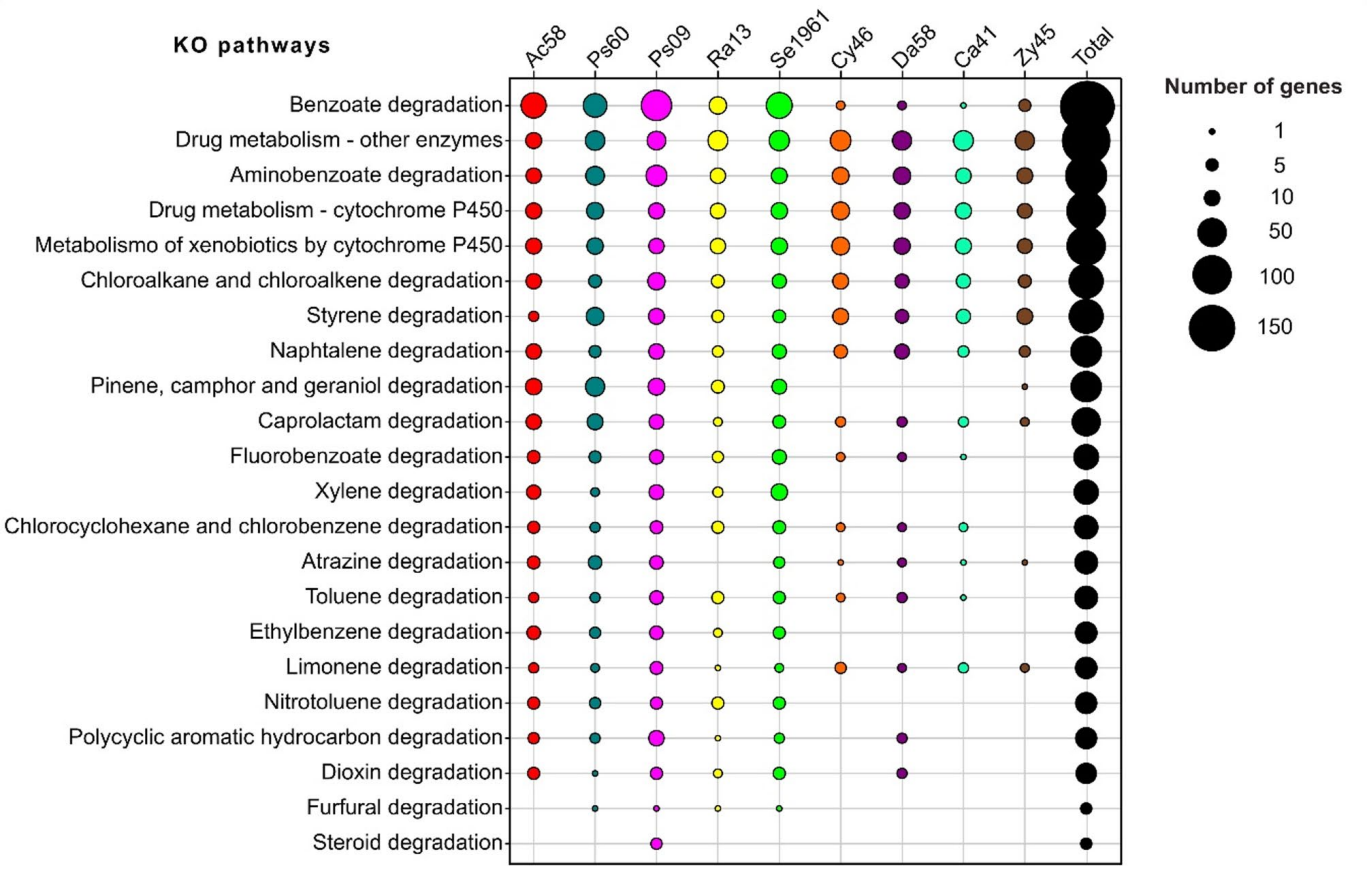
Bubble plot of absolute abundance of genes for KEGG orthology (KO) pathways related to the degradation of xenobiotics. The chart shows the number of genes from dominant microorganisms of *D. rhizophagus* gut (X-axis) annotated in 22 KO pathways of xenobiotic biodegradation and metabolism, limonene degradation, and pinene, camphor and geraniol degradation (Y-axis). The bacteria and yeasts showed a total of 1,293 genes identified whether differential or exclusive for some of these KO pathways reported

A total of 28,894 genes of gut core members of *D. rhizophagus* were mapped to 439 KO pathways (Supplemental Table 3). Within these, 22 pathways were related to xenobiotic degradation with 1,293 genes from both bacteria and yeasts. However, the number of genes associated with such pathways varied within and among bacteria and yeasts (Fig. 2).

The following 10 KO pathways highlighted the presence of xenobiotic-degradation genes: Benzoate degradation (ko00362), Drug metabolism-other enzymes (ko00983), Aminobenzoate degradation (ko00627), Drug metabolism-cytochrome P450 (ko00982), Metabolism of xenobiotics by cytochrome P450 (ko00980), Chloroalkane and chloroalkene degradation (ko00625), Styrene degradation (ko00643), Naphthalene degradation (ko00626), Caprolactam degradation (ko00930), and Limonene degradation (ko00903). Also, benzoate degradation, aminobenzoate degradation, drug metabolism - other enzymes, styrene degradation, caprolactam degradation, and naphthalene degradation presented the highest number of different enzymes annotated in these KO pathways (> 10 KO enzyme entries; Supplemental Table 3).

Seven KO pathways were differentially represented by genes belonging to some bacteria and yeasts: Pinene, camphor and geraniol (ko00907) degradation with the highest number of enzymes (12 KO enzymes entries), Fluorobenzoate degradation (ko00364), Chlorocyclohexane and chlorobenzene degradation (ko00361), Atrazine degradation (ko00791), Toluene degradation (ko00623), Polycyclic aromatic hydrocarbon degradation (ko00624), and Dioxin degradation (ko00621). Conversely, five KO pathways were supported exclusively by bacterial genes: Xylene degradation (ko00622) with the highest number of enzymes (17 KO enzymes entries), Ethylbenzene degradation (ko00642), Nitrotoluene degradation (ko00633), Furfural degradation (ko00365), and Steroid degradation (ko00984) (Fig. 2).

**Fig. 3:**
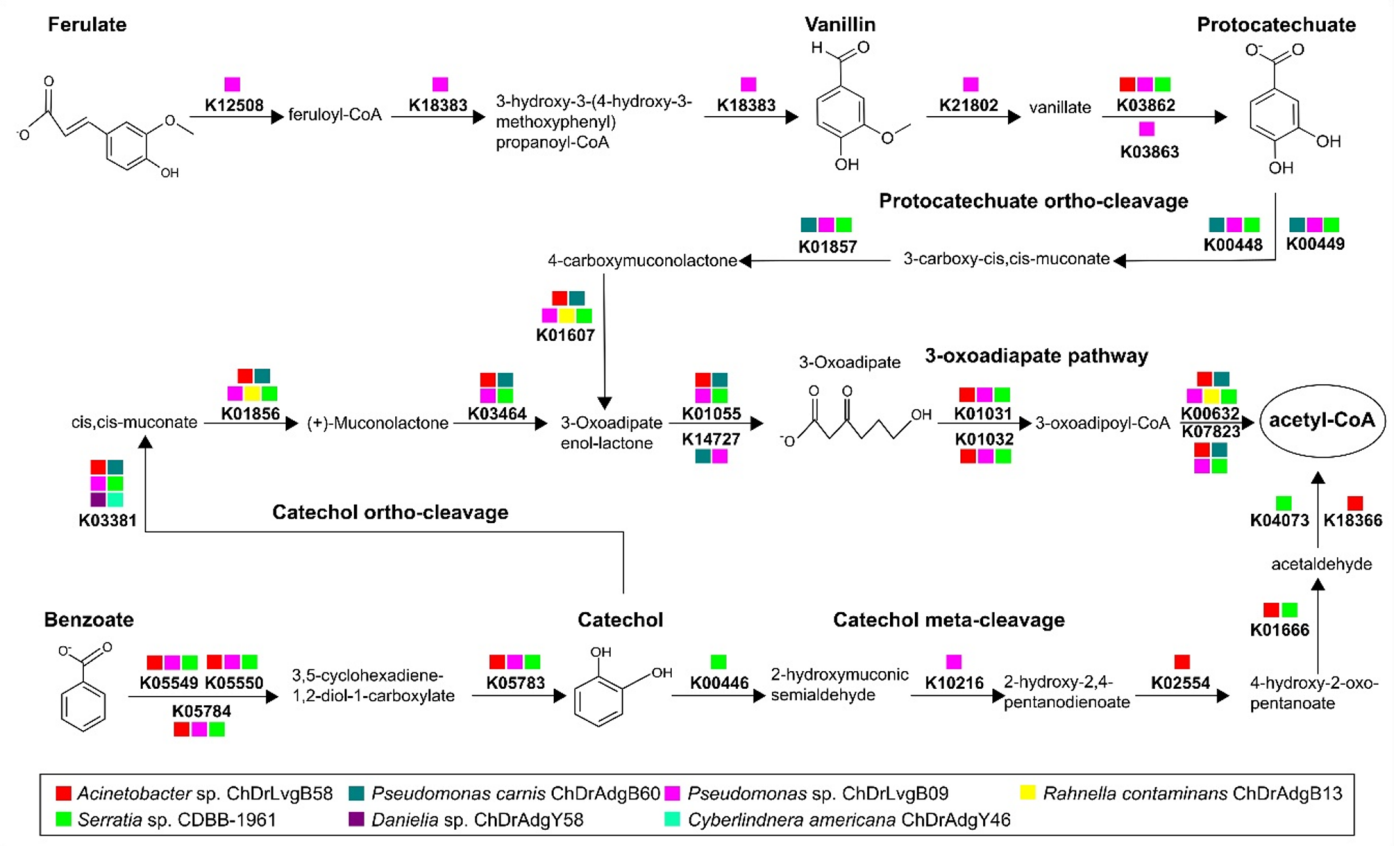
Degradation pathways of the aromatic compounds ferulate, vanillin and benzoate. The scheme shows 28 KO-enzymes from bacterial and yeast genes annotated into the degradation of aromatic compounds, including oxidation, protocatechuate ortho-cleavage, catechol meta-cleavage, catechol ortho-cleavage, and 3-oxoadiapate pathways. The description of KO enzymes can be found in Supplemental Table 3

Five complete modules (gene and reaction set) in aromatic compounds degradation were recovered: Benzoate degradation (M00551; benzoate → methylcatechol), Anthranilate degradation (M00637; anthranilate → catechol), Catechol ortho-cleavage (M00568; catechol → 3-oxoadipate), Catechol meta-cleavage (M00569; catechol → acetyl-CoA/4-methylcatechol → propanoyl-CoA) and Phenylacetate degradation (M00878; phenylaxetate → acetyl-CoA/succinyl-CoA) (Supplemental Table 3). Among these modules, 28 KO-enzymes related to the catabolism of ferulate, vanillin and benzoate were found following the protocatechuate ortho-cleavage, catechol meta-cleavage, catechol ortho-cleavage, and 3-oxoadiapate degradation pathways (Fig. 3).

Additional KO-enzymes not integrated in modules and related to terpene metabolism were found, such as aldehyde dehydrogenase (NAD+) [EC:1.2.1.3] and epsilon-lactone hydrolase [EC:3.1.1.83], annotated to limonene degradation; the enoyl-CoA hydratase [EC:4.2.1.17], involved in α-pinene degradation; and 11 KO-enzymes linked with nerol/geraniol/citronellol degradation (Supplemental Table 3).

**Fig. 4:**
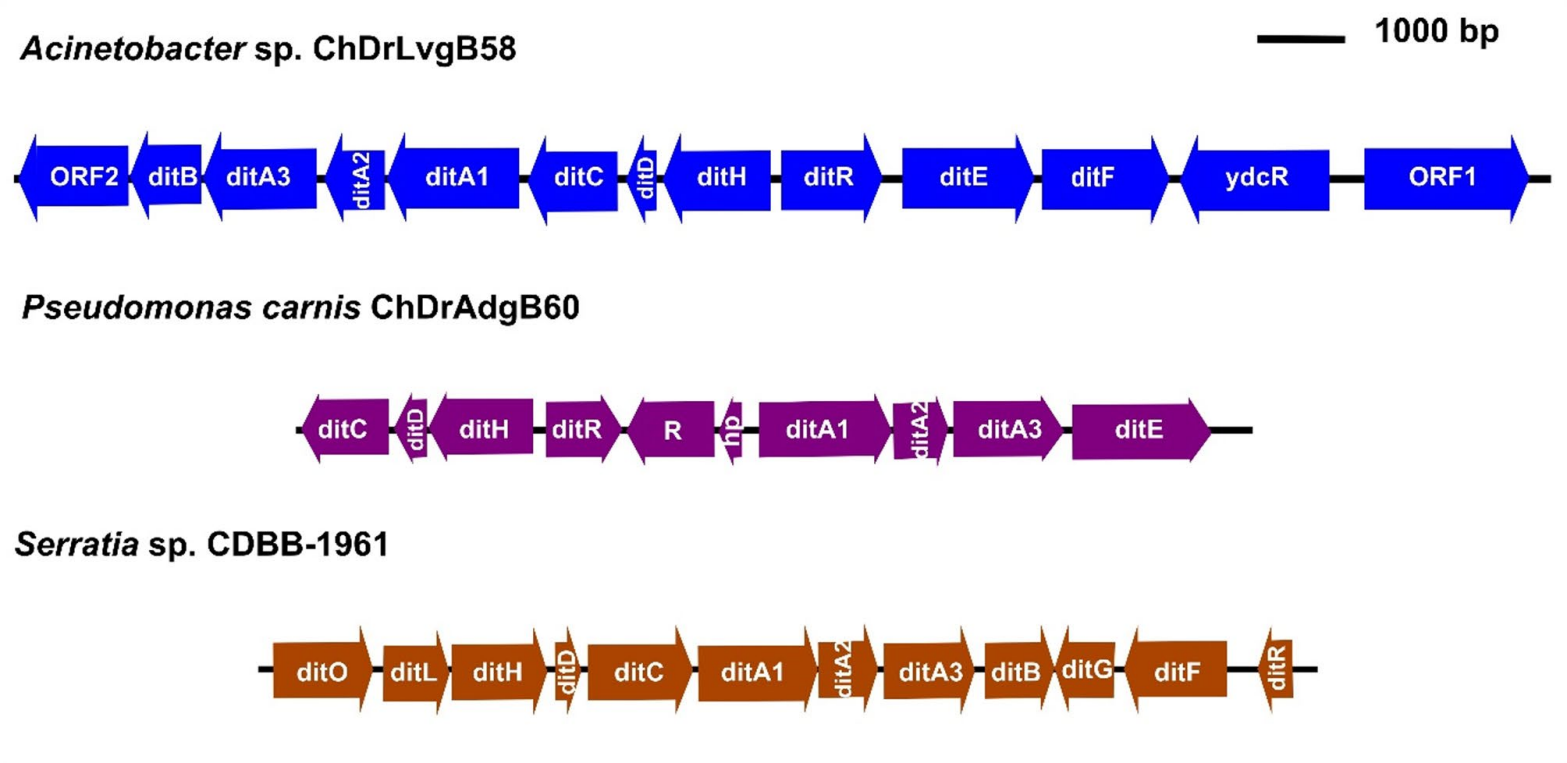
Clusters of *dit* genes in bacteria of gut core microbiome. The clusters show symbols, orientations, sizes and positions of *dit* genes in the genomes of each bacterium. Fifteen different *dit* genes were recovered among the clusters of *Acinetobacter* sp. ChDrLvgB58, *P. carnis*. ChDrAdgB60, and *Serratia* sp. CDBB-1961 strains: α- and β-subunit of the ring-hydroxylating dioxygenase (ditA1 and ditA2), Ferredoxin component of ring-hydroxylating dioxygenase (ditA3), Dehydrogenase/reductase (ditB and ditG), cleavage dioxygenase (ditC), Isomerase/decarboxylase (ditD and ditH), Permease of MFS (ditE and ORF2), Sterol carrier-like protein (ditF), IclR-type transcription regulator (ditR), AB hydrolase (ditL), Thiolase (ditO), and CoA ligase (ORF1). Also unrelated *dit* genes, such as ydcR (repressor), R (regulator) and hp (hypothetical protein) were among the clusters

Complete clusters of genes in tandem associated with diterpene degradation (putative *dit* genes) were identified in the bacteria *Acinetobacter* sp. ChDrLvgB58, *P. carnis* ChDrAdgB60, and *Serratia* sp. CDBB-1961 (Fig. 4). The number of genes in each cluster ranged from 12 for *Acinetobacter* and *Serratia* strains, and eight in *Pseudomonas*, including genes for ligases, reductases, dioxygenases, decarboxylases, permeases, hydrolases, thiolases and regulators. This highlighted the presence of 15 genes encoding for the CoA ligase (ORF1), α and β subunits of the ring-hydroxylating dioxygenase (ditA and ditA2), Isomerase/decarboxylase (ditH), Dehydrogenase/reductase (ditG), Sterol carrier-like protein (ditF), IclR-type transcription regulator (ditR), Permease of the major facilitator superfamily (ditE), Isomerase/decarboxylase (ditD), cleavage dioxygenase (ditC), Dehydrogenase/reductase (ditB), Ferredoxin component of ring-hydroxylating dioxygenase (ditA3), Permease of the major facilitator superfamily (ORF2), AB hydrolase (ditL), and Thiolase (ditO).

### Protein families associated with the detoxification process

**Fig. 5:**
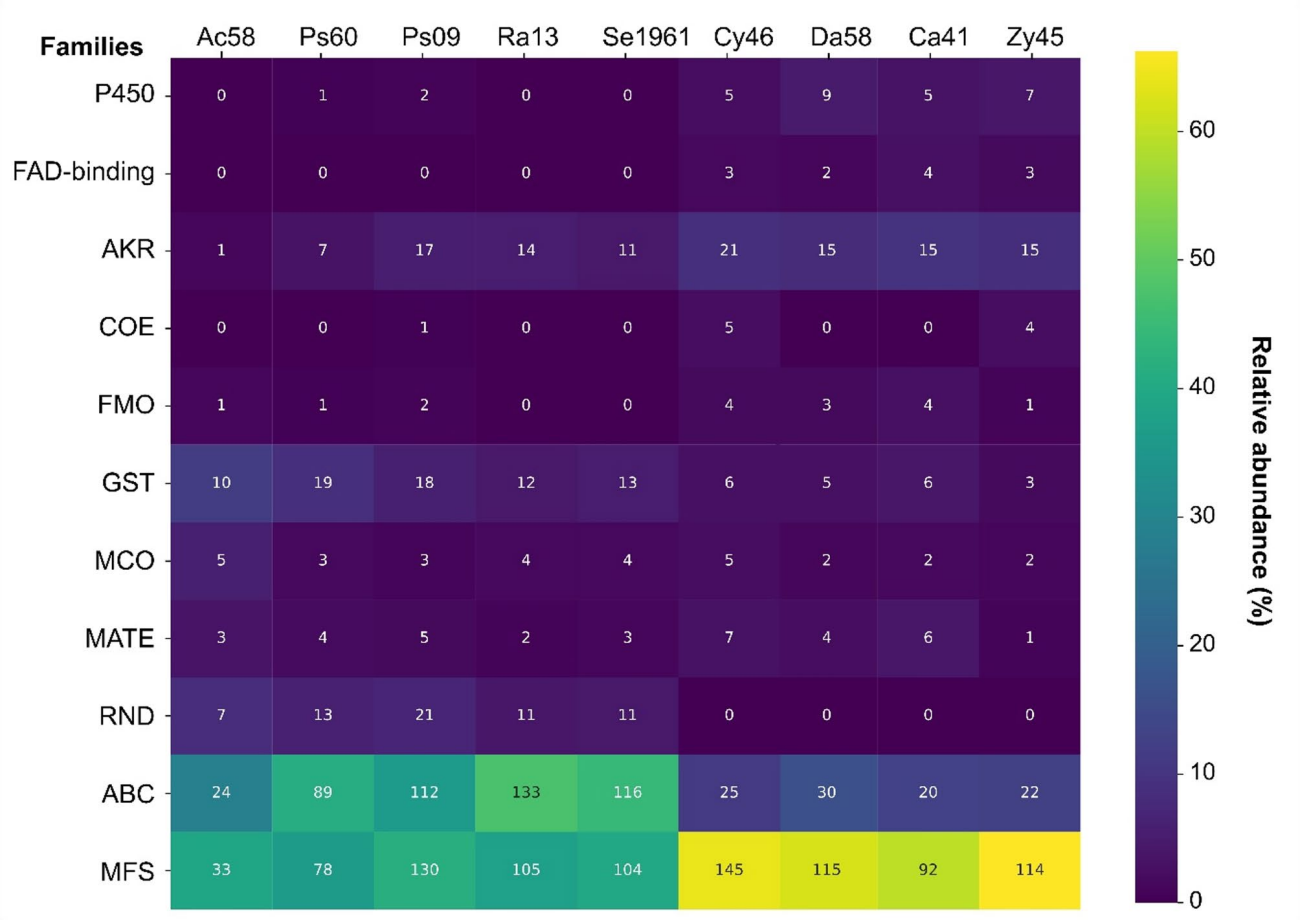
Heatmap of enzyme and transporter families annotated in Pfam. The graph shows the relative (% in colors) and absolute (number within boxes) abundance of genes annotated to protein families associated with xenobiotic detoxification in all microorganisms. P450 = Cytochrome P450, FAD-binding = FAD binding domain enzymes, AKR = Aldo/keto reductase family, COE = Carboxylesterase family, FMO = Flavin-binding monooxygenase, GST = Glutathione S-transferase, MCO = Multicopper oxidase, MATE = Multi-antimicrobial extrusion protein, RND = Resistance-Nodulation-Division, ABC = ATP-binding cassette transporters, MFS = Major Facilitator Superfamily

From all microorganisms, a total of 1,890 genes belonging to 11 protein families associated with the detoxification process were identified (Supplemental Tables 4 and 5). Four of these families were MDR transporters (MFS, ABC, RND and MATE) and seven enzymatic families (GST, AKR, P450, FAD-binding, COE, FMO, and MCO). The ABC and MFS transporters were the best represented in these microorganisms; ABC transporters were particularly more abundant in bacteria (> 30% total genes) than in yeasts, while MFS genes showed an inverse relationship (> 60% in yeast genes). The MATE and RND transporters had < 10% abundance, the former was present in all microorganisms, and the latter only in bacteria (Fig. 5).

Within enzymatic families, the GTSs and AKRs were highlighted by their abundance in bacteria (10%−19%) and yeasts (15%−21%). The families FMO, FAD-binding, COE, MCO, and P450 had low abundance (< 10%) in all microorganisms, and some were recovered from specific strains, such as FAD-binding enzymes identified only in yeasts, and cytochromes P450 present exclusively in *Pseudomonas* strains and all yeasts (Fig. 5).

### ABC transporters

**Fig. 6:**
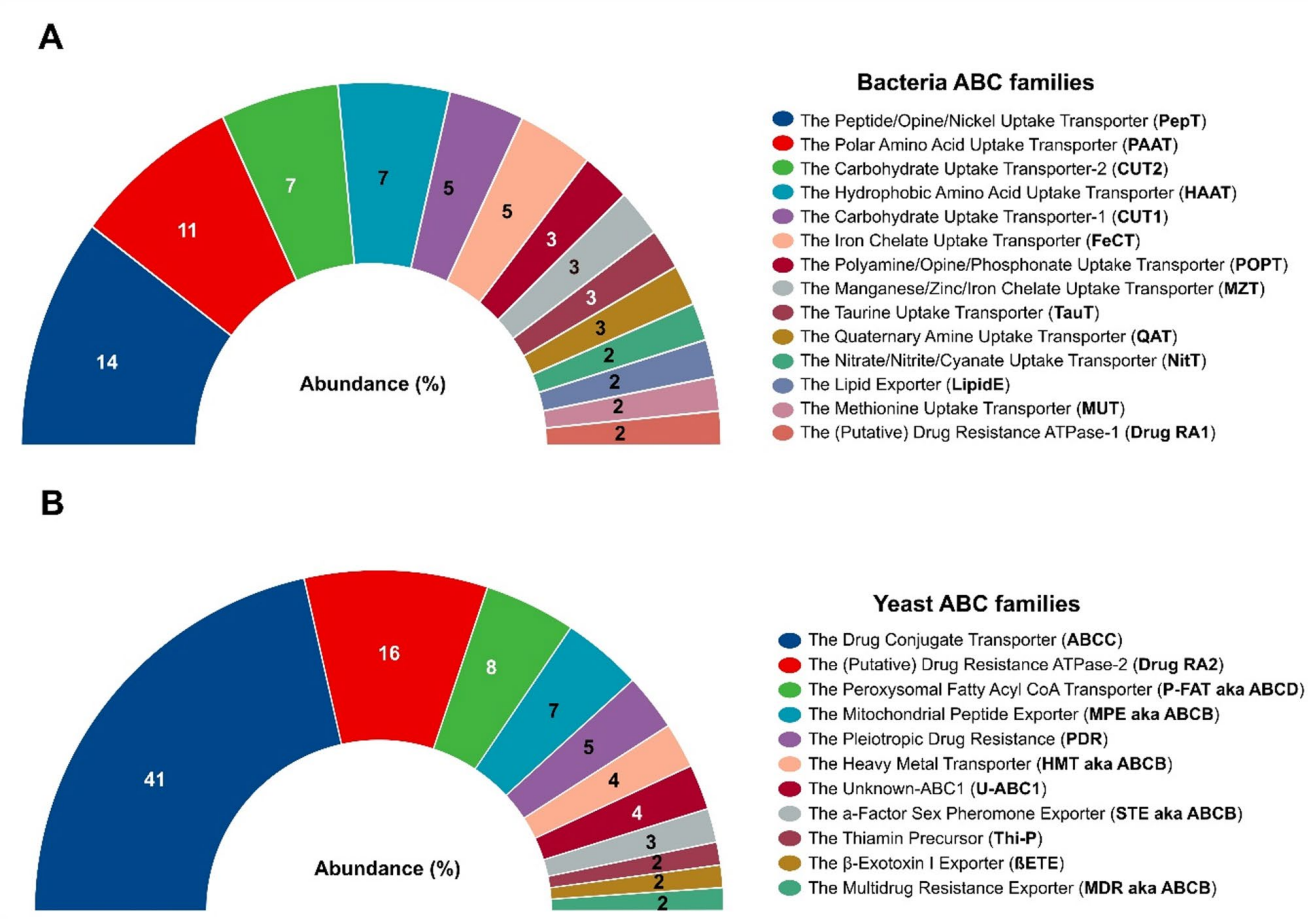
Abundance of the ABC subfamilies. The graphs show the relative abundance (%) of genes of the ABC families annotated in TCDB database with percentages ≥ 2% of bacteria (**A**) and yeasts (**B**)

Bacterial ABC transporters were represented by 474 genes, whose length varied from 130 to 888 amino acids (aa) with molecular weight from 14.5 to 97 kDa, isoelectric point (pIs) from 4.5 to 10.7, and subcellular location mainly in the cytoplasmic membrane (Supplemental Table 4). These genes were classified into 55 different subfamilies of ABC transporters, 14 of which accounted for 70% of the total number of genes for these transporters (Fig. 6A). The greatest percentage of genes were found in the subfamilies Peptide/Opine/Nickel Uptake Transporter (PepT, 68 genes), Polar Amino Acid Uptake Transporter (PAAT, 51 genes), The Carbohydrate Uptake Transporter-2 (CUT2, 36 genes), Hydrophobic Amino Acid Uptake Transporter (HAAT, 34 genes), Carbohydrate Uptake Transporter-1 (CUT1, 23 genes) and Iron Chelate Uptake Transporter (FeCT, 23 genes).

In yeasts, 97 genes were associated with ABC transporters, whose length varied from 90 to 1692 aa with a molecular weight between 10.14 and 189.61 kDa, pIs from 5.23 to 10.01, and subcellular location in vacuoles, cytoplasm, mitochondria, nucleus, cell membrane and peroxisomes (Supplemental Table 5). These genes were classified into 15 subfamilies, 11 of which clustered to 96% of the ABC genes (Fig. 6B). The subfamilies Drug Conjugate Transporter (ABCC, 40 genes), (Putative) Drug Resistance ATPase-2 (Drug RA2, 16 genes), and Peroxisomal Fatty Acyl CoA Transporter (P-FAT aka ABCD, 8 genes) contained the greatest number of genes in yeast ABC transporters.

### MFS transporters

**Fig. 7:**
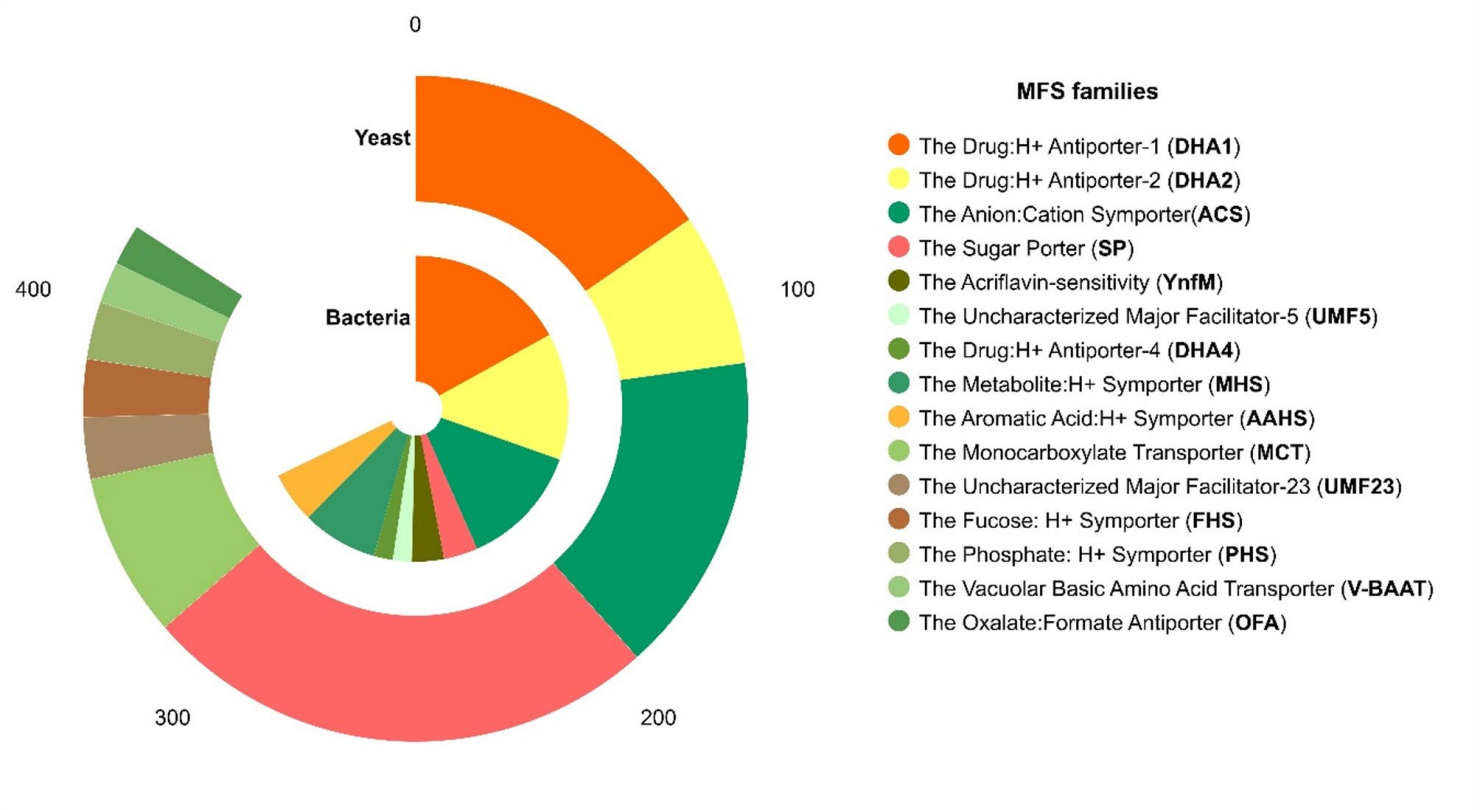
Abundance of the MFS subfamilies. The absolute abundance (number of genes) of MFS subfamilies in bacteria and yeast is represented in the chart. The families annotated in TCDB database with percentages ≥ 2% are shown

A total of 450 and 466 MFS genes were identified in bacteria and yeasts, respectively. The length of bacterial genes varied from 71 to 722 aa, with a molecular weight between 7.95 and 78.74 kDa, pI values from 4.8 to 12, and cellular localization in the cytoplasmic membrane (Supplemental Table 4). MFS bacterial genes were classified into 38 subfamilies including Drug: H + Antiporter-1 (12 Spanner) (DHA1, 85 genes), Drug: H + Antiporter-2 (14 Spanner) (DHA2, 67 genes), Anion: Cation Symporter (ACS, 65 genes), Metabolite: H + Symporter (MHS, 40 genes), Aromatic Acid: H + Symporter (AAHS, 27 genes), Sugar Porter (SP, 18 genes), Acriflavin-sensitivity (YnfM, 17 genes), Uncharacterized Major Facilitator-5 (UMF5, 10 genes) and Drug: H + Antiporter-4 (DHA4, 10 genes), and 75.3% of the total genes were grouped (Fig. 7).

The length of yeast MFS transporters varied from 168 to 1063 aa with molecular weight ranging from 18.53 to 118.44 kDa, pIs from 4.51 to 9.44, and cell location in the cell membrane, vacuoles, endoplasmic reticulum, and Golgi apparatus (Supplemental Table 5). These genes were classified into 19 MFS subfamilies, which included transporters SP (126 genes), ACS (78 genes), DHA1 (77 genes), Monocarboxylate Transporter (MCT, 40 genes), DHA2 (37 genes), Uncharacterized Major Facilitator-23 (UMF23, 15 genes), Fucose: H + Symporter (FHS, 14 genes), Phosphate: H + Symporter (PHS, 14 genes), Vacuolar Basic Amino Acid Transporter (V-BAAT, 10 genes), and Oxalate: Formate Antiporter (OFA, 10 genes), which clustered 90.3% of the total genes identified (Fig. 7).

### MATE and RND transporters

MATE transporters were identified in bacteria and yeasts by the presence of 17 and 18 genes, respectively. The length of bacterial genes varied from 390 to 483 aa, with molecular weight ranging from 43.77 to 52.26 kDa, pIs from 8.37 to 10.96, and predicted subcellular location in the cytoplasmic membrane. Bacterial MATE genes were classified into nine subfamilies as follows: DNA damage-inducible protein F (DinF, 3 genes), Probable multidrug resistance protein (YoeA, 3 genes), Drug: H + antiporter pump (PmpM, 2 genes), Quinolone: H + antiporter (EmmdR, 2 genes), Multidrug-resistance efflux pump (MdtK-NorE-YdhE, 2 genes), Drug (norfloxacin, polymyxin B) resistance efflux pump (NorM, 2 genes), H+-coupled multidrug efflux pump (AbeM, 1 gene), Ciprofloxacin efflux pump (AbeM4, 1 gene), and MATE exporter protein (1 gene) (Supplemental Table 4). Conversely, the 18 yeast MATE genes were classified only into Ethionine resistance protein (ERC1). The length of these genes varied from 278 to 645 aa, with molecular weights between 30.7 and 71.16 kDa, pIs from 4.8 to 8.23, and predicted location in vacuole, cell membrane and endoplasmic reticulum (Supplemental Table 5).

Sixty-three bacterial genes were associated with RND transporters (Supplemental Table 4), and no gene of this type was identified in yeast. The length of genes varied from 206 to 1,073 aa with molecular weight between 22.23 and 115.8 kDa, pIs from 4.7 to 10.4, and a predicted location in the cytoplasmic membrane. The bacterial RND genes were classified into the families Hydrophobe/Amphiphile Efflux-1 (HAE1, 53 genes), Putative Nodulation Factor Exporter (NFE, 5 genes), and Heavy Metal Efflux (HME, 5 genes).

### AKR enzymes

In bacteria and yeasts, 50 and 66 AKR genes were identified, respectively. The length of bacterial genes varied from 124 to 353 aa, with molecular weight between 13.6 and 39.43 kDa, pIs from 4.64 to 7.07, and a predicted location in the cytoplasm. The bacterial AKR were categorized into 18 classes, including AKR_Tas-like (6 genes), AKR_EcYajO-like (4 genes), AKR13A_13D (4 genes), AKR13C1_2 (4 genes), 2,5-diketo-D-gluconate reductase A [EC:1.1.1.346] (dkgA, 4 genes), 2,5-diketo-D-gluconate reductase B [EC:1.1.1.346] (dkgB, 4 genes), and L-glyceraldehyde 3-phosphate reductase [EC:1.1.1.-] (yghZ, 4 genes) which grouped 60% of the total genes identified as AKR (Supplemental Table 4). The length of yeast AKR genes ranged from 163 to 395 aa having molecular weight from 18.24 to 44.82 kDa, pIs from 4.92 to 8.08, and cellular location in the cytoplasm and nucleus (Supplemental Table 5). Yeast AKR genes were categorized into 15 classes, including the aryl-alcohol dehydrogenase (17 genes), glycerol 2-dehydrogenase (NADP+) [EC:1.1.1.156] (GCY1, 8 genes), AKR1-5-like (6 genes), D-xylose reductase [EC:1.1.1.307 1.1.1.307 1.1.1.431] (5 genes), D-arabinose 1-dehidrogenase [EC:1.1.1.117] (ARA1, 5 genes), and pyridoxine 4-dehidrogense [EC:1.1.1.65] (5 genes) clustered in 69.7% of the total AKR genes identified (Supplemental Table 5).

### COE, FAD-binding, and FMO enzymes

Only 10 COE genes were identified, one in bacteria and nine in yeast. The bacterial COE was classified as periplasmatic para-nitrobenzyl esterase [EC:3.1.1.-] (pnbA). The length of the bacterial gene was 516 aa, with a molecular weight of 55.05 kDa, pIs of 6.5, and periplasmic location. The yeast COEs were subclassified as type-B carboxylesterase lipases, their length varied from 526 to 564 aa, with molecular weight from 57.72 to 61.98 kDa, pIs from 4.31 to 6.57, and a predicted location in the cytoplasm, extracellular, endoplasmic reticulum, vacuoles, and nucleus.

The FAD-binding enzymes were represented by 12 genes found only in yeast. These genes were subclassified as NADPH-ferrihemoprotein reductase [EC:1.6.2.4] (CPR, 5 genes), sulfite reductase (NADPH) flavoprotein alpha-component [EC:1.8.1.2] (MET10, 4 genes), and NADPH-dependent diflavin oxidoreductase 1 (TAH18, 3 genes). These genes ranged from 586 to 1108 aa, with molecular weight between 67.36 and 120.78 kDa, pIs from 4.94 to 6.96, and cellular location in the endoplasmic reticulum and cytoplasm.

Four bacterial and 12 yeast FMO genes were identified. The lengths of these bacterial genes varied from 429 to 512 aa, with molecular weight between 47.18 and 54.9 kDa, pIs from 5.89 to 9.48, and a cytoplasmic membrane and cytoplasm location. Bacterial FMOs were classified as cyclohexanone monooxygenase [EC:1.14.13.22] (2 genes), a putative flavoprotein involved in K + transport (1 gene), and L-ornithine N (5)-monooxygenase-related (1 gene). The length of yeasts´s FMOs ranged from 461 to 569 aa, with molecular weight from 51.54 to 65.24 kDa, pIs from 5.35 to 9.09, and predicted location in the cytoplasm and nucleus. Yeast FMOs were classified as dimethylaniline monooxygenase (FMO1, 8 genes), thiol-specific monooxygenase (2 genes), pyridine nucleotide-disulphide oxidoreductase (1 gene), and cyclohexanone monooxygenase [EC:1.14.13.22] (1 gene).

### GST and MCO enzymes

Seventy-two bacterial and 20 yeast GSTs genes were identified. The GST length of bacteria varied from 93 to 331 aa, with molecular weight between 10.74 and 37.59 kDa, pIs from 4.62 to 9.89, and the cytoplasm was the main predicted subcellular localization. Bacterial GSTs were classified into 18 classes (Supplemental Table 4) highlighted by their abundance GstA (14 genes), GST yfcF (7 genes), GSH-dependent disulfide-bond oxidoreductase [EC:1.8.4.-] yfcG (6 genes), glutathionyl-hydroquinone reductase [EC:1.8.5.7] yqjG (GS-HQR, 6 genes), and GST yliJ (6 genes). The length of yeast GSTs varied from 217 to 347 aa, with molecular weight between 24.61 and 39.82, pIs from 5.31 to 8.65, and predicted location in the cytoplasm, nucleus and cell peroxisome. The GSTs of yeasts were classified into eight classes (Supplemental Table 5), with the most representative being GS-HQR ECM4 (4 genes), GST1 (4 genes), URE2 (4 genes), and GST2 (3 genes).

A total of 19 and 11 MCO genes were identified in bacteria and yeasts, respectively. Bacterial MCO were subclassified as laccases (yfiH, 8 genes), copper resistance protein (copA, 4 genes), cuproxidase [EC:1.16.3.4] (cueO, 2 genes), multicopper oxidase (cumA, 2 genes), suppressor of ftsI (sufI, 2 genes), and L-ascorbate oxidase [EC:1.10.3.3] (1 gene). The length of bacterial MCO varied from 205 to 630 aa with molecular weight between 22.8 and 71.09 kDa, pIs from 8.68 to 5.15, and located in the cytoplasm and periplasm (Supplemental Table 4). Yeast MCO were classified as iron transport multicopper oxidases (FET3, 8 genes), ascorbase (1 gene), laccase (1 gene), and uncharacterized MCO (1 gene). Their length varied from 610 to 663 aa, with molecular weight between 69 and 76 kDa, pIs from 4.21 to 5.5, and predicted cellular localization in the cell membrane, endoplasmic reticulum, vacuole, Golgi apparatus, and extracellular (Supplemental Table 5).

### Cytochromes P450

Three enzymes belonging to the cytochrome P450 family were determined only in *Pseudomonas* strains. These cytochromes P450 were clustered into three phylogenetic groups: (1) CypX (Terpene synthase family 2), (2) CYP105-like, and (3) CYP105T1 (Supplemental Fig. 1, Additional file 1). The length of these P450s varied from 372 to 741 aa, with molecular weight between 41.91 and 81.8 kDa, pIs from 5.34 to 6.05, and predicted location in the cytoplasm (Supplemental Table 4).

In yeast, twenty-six P450 were identified, with lengths varying from 481 to 574 aa, and molecular weight between 55.75 and 65.83, pIs from 5.41 to 9.12, and predicted subcellular location in the endoplasmic reticulum (Supplemental Table 5). These cytochrome P450 were phylogenetically grouped into seven families: CYP51 (ERG11) and CYP61 (ERG5) identified in all yeast strains; CYP504 (PHAC) in all yeasts strains, except *Danielia* sp. ChDrAdgY58; CYP52 (ALK) linked to *Danielia* sp. ChDrAdgY58 and *Zygoascus* sp. ChDrAdgY45; CYP56 (DIT) present in *C. americana* ChDrAdgY46 and *Danielia* sp. ChDrAdgY58, and CYP5216/CYP52XX present in all yeast strains, except *Zygoascus* sp. ChDrAdgY45 (Supplemental Fig. 2, Additional file 1).

## Discussion

In this study, we investigated the potential detoxification capacity of the dominant members of the gut core bacteriome and mycobiome from bark beetle *D. rhizophagus*. The BUSCO results showed that more than 90% that of the total number of genes predicted (3,427 to 6,276 CDs) in each strain were recovered as complete unicopy orthologues, which gives certainty to their functional annotation [56]. The gene functional annotation showed an unequal association in the number and type of genes (Fig. 1), including those associated with the three phases of detoxification (Supplemental Table 6). This suggested that the strains have their own metabolic capacities, but are also complementary and perhaps compensatory, during xenobiotic detoxification.

### Phase I of detoxification

From genes annotated in all these microbes, 149 enzymes corresponded to detoxification phase I (Supplemental Table 6). Among the most abundant enzymes highlighted are oxidoreductases followed by hydrolases, lyases, and isomerases, including cytochromes P450 (P450), aldehyde dehydrogenase (NAD+) enzymes, COEs, FMOs, and FAD-binding reductases, among others. These enzymes are responsible for the oxidation, reduction or hydroxylation of compounds derived from the metabolism or acquired across the environment, such as terpenes and terpenoid compounds [70].

Our findings showed that the diversity and relative abundance of these genes is higher in bacteria than in yeast strains (Supplemental Table 6), suggesting the active participation directly or indirectly of bacteria in the xenobiotic transformation. In vitro studies performed with bacteria isolates (e.g., *Pseudomonas*, *Serratia*, *Rahnella*) from bark beetles or their galleries have demonstrated that they are capable of tolerating and degrading monoterpenes [21, 22].

The presence of P450 genes of the subfamilies CYP105T1, CYP105-like and CYPX only in *Pseudomonas* strains (Supplemental Fig. 1, Additional file 1), corroborates other studies conducted with environmental bacteria which showed that CYP450 are related to terpenes metabolism [71], such as CYP101A1 of *Pseudomonas putida* and CYP105D1 of *Streptomyces griseus* involved in the oxidation of camphor [71, 72].

The lack of P450 genes in *Acinetobacter*, *Serratia* and *Rahnella* is a property shared with other Gammaproteobacteria, such as *Escherichia*, *Salmonella*, *Shigella*, *Citrobacter*, and *Raoultella* [73]. Nevertheless, the absence of these enzymes in strains associated with the *D. rhizophagus* gut indicates that the degradation and transformation of terpenes or oxygenated-terpenes could be carried out by other enzymes or through other metabolic pathways. This lack of P450 genes is not a characteristic of all *Acinetobacter*, *Serratia* and *Rahnella* species, as CYP105 and CPY1414 have been reported in *Serratia plymuthica* and *Serratia rubidaea*, which are involved in the synthesis of ß-lactam antibiotics [73]. The CYP116B in *Acinetobacter radioresistens* catalyzes the terminal hydroxylation of alkanes [74]. Also, CYP1415 has been recorded in *Rahnella aquatilis* but its function is unknown [73].

In contrast, our results showed that the P450 genes encoding for CYPs belong to clades 2 (CYP52) and 10 (CYP504) [75, 76] in all yeast strains, except in *Danielia*, in which the CYP504 is absent (Supplemental Fig. 2, Additional file 1). These cytochromes, together with the redox partner NADPH-ferrihemoprotein reductase [EC:1.6.2.4] (CPR) (Supplemental Table 5), comprise the class II CYP system [77]. In yeasts associated with bark beetles, the functional role of these cytochromes is unknown, but in environmental yeasts (e.g., *Candida* and *Yarrowia*), CYP52 has been associated with the oxidation of alkanes [75]. The CYP504 (*phac protein)* has been identified in yeast and filamentous fungi suggesting its participation in phenylacetate catabolism [65]. Particularly, the overexpression of CYP504 in *Grosmannia clavigera*, an ectosymbiont associated with *Dendroctonus ponderosae*, is related to the metabolism of fatty acids and phenolic compounds [78]. Similarly, the functional role of cytochromes P450 in the metabolism of terpenes to produce different oxygenated compounds has been evidenced in some bark beetles of the genus *Dendroctonus* [79, 80]. Therefore, we hypothesized that both *D. rhizophagus* and yeasts could help to overcome the hos’t defenses through the oxidation of tree xenobiotics by cytochromes P450 and that other bacterial oxidoreductases could continue the biotransformation of the derived compounds.

Flavin-binding Monooxygenases (FMO) associated with detoxification phase I were classified as cyclohexanone monooxygenase [CHMO, EC: 1.14.13.22] in bacteria *Acinetobacter* and *Pseudomonas*, as well as in the yeast *Danielia*. This enzyme, along with cyclopentadecanone monooxygenase and monocyclic monoterpene ketone monooxygenase, carries out Baeyer-Villiger-type reactions (BVMO), catalyzing the oxidation of cyclic ketones to esters [81]. This is the first report of CHMO in bacteria and yeast associated with insect gut. These enzymes have been involved in the transformation of cyclic terpenoids into lactones in environmental bacteria, yeast, and filamentous fungi [82]. Particularly in the filamentous fungi *G. clavigera*, the CHMO is essential for fungal growth in the presence of limonene [83]. The presence of CHMOs in bacteria and yeast associated with the gut of bark beetle, suggested the existence of alternative or complementary ways associated with the transformation of cyclic monoterpenes present in the phloem or derived from insect metabolism during the detoxification process. The presence of FMO1 in yeasts (Supplemental Table 5) suggests a possible role in the repair of cellular damage produced by the action of the tree´s compounds as it has been reported in *Saccharomyces cerevisiae* [84]. This is because these enzymes oxidize thiol compounds that maintain the intracellular redox regulation and the correct folding of protein [85]. While FMOs in vertebrates play a fundamental role in xenobiotic detoxification [86], the presence and function of these enzymes in bark beetles and their symbionts are limited [83, 87, 88] so we suggest that their functions should be tested.

Our findings revealed that the oxidoreductases most abundant in all bacteria and yeast were the aldehyde dehydrogenases (NAD+) [EC:1.2.1.3; ALDH] and alcohol dehydrogenases [EC:1.1.1.1; ADH], which are associated with the metabolism of limonene and diverse xenobiotics (Supplemental Table 6). Although the mechanism of terpene degradation by these enzymes is undocumented in bark beetle symbionts, in various *Pseudomonas* species isolated from soil, it has been demonstrated that both enzymes oxidize acyclic terpenes such as geraniol and citronellol [89, 90]. The possibility that both ADH and ALDH act in the biotransformation of oxygenated terpenes cannot be ruled out since in vitro experimental assays performed with *Dendroctonus* symbionts have demonstrated their ability to transform verbenols to verbenone, an anti-aggregation pheromone of many bark beetles [27, 28]. In addition, the overexpression of ALDH and ADH genes in *Cyberlindnera americana* and *Leptographium qinlingensis* has been documented after these fungal symbionts were stimulated with different terpenes [44, 91]. Furthermore, in *Enterobacter xiangfangensis* a symbiont of the *D. valens’s* gut, it has been demonstrated that ALDH1 can transform the cis-verbenol to verbenone [45]. It is noteworthy that ALDHs are present in genomes of some *Dendroctonus*-bark beetles [92, 93], and they are overexpressed after stimulating insects with monoterpenes (unpublished data). This evidence suggests that these enzymes, both from insects and their intestinal microorganisms, could play a relevant role in the detoxification of monoterpenes.

Few genes belonging to carboxylesterases (COE) and laccases (MCO) phase I are present in the genomes of some bacteria and yeast of *D. rhizophagus´s* gut (Supplemental Table 6). Both COE and MCO have been identified in diverse microorganisms and insects and are involved in the detoxification processes of a wide variety of xenobiotics (e.g. polyphenols, aromatic compounds, lignin derivatives, organophosphates, carbamates and pyrethroids) [94, 95]. For example, the laccase enzymatic activity was demonstrated in the symbiont fungus *L. qinlingensis* of *Dendroctonus armandi* when the fungus was grown with sawdust [91]. To our knowledge, no study has demostrated the functional role of COEs in the detoxification process in bark beetles and their symbionts, despite being overexpressed in *Dendroctonus* species stimulated with terpenes [80, 96]. Seemingly the functional activity of these enzymes could be related to a compensatory metabolic and physiological process triggered indirectly by the action of diverse aromatic compounds, allelochemicals or lignocellulose derivatives from trees.

Notably, our annotations show *dit* gene clusters involved in diterpene degradation in *Acinetobacter*, *Pseudomonas* and *Serratia* bacteria (Fig. 4). The number of *dit* genes in these clusters is variable, twelve in *Acinetobacter* and *Serratia*, and eight in *Pseudomonas*. The individual capacity of some enzymes encoded by *dit* genes to degrade diterpenes have been demonstrated in *Pseudomonas*, *Cupriavidus*, and *Burkholderia* strains isolated from water and soil [97, 98]. There is a variable number of genes in clusters among species of the same genus. For example, *Pseudomonas abietaniphila* has 19 *dit* genes [97], compared with the number found in this study for the *Pseudomonas* strain. However, not all of them are directly involved in the catabolism of diterpenes, since the enzymes encoded by the ditA1, ditA3, ditC, ditE, ditF, and ditH genes are fundamental for the degradation of abietic acid [97]. These essential genes are present in *Acinetobacter*, *Serratia* and *Pseudomonas* associated with *D. rhizophagus* gut (Fig. 4). The *dit* genes have also been identified from metagenomic assays of the gut bacterial communities of the bark beetle *D. ponderosae* and the pine weevil *Hylobious abietis* [42, 99].

A large set of genes encoding for dehydrogenases, hydratases, monooxygenases, intradiol dioxygenases and extradiol dioxygenases is notable in the annotation of bacterial genomes, as well as in the yeasts *Danielia* sp. and *C. americana* (Supplemental Table 6). These enzymes are involved in the oxidation and ring cleavage of aromatic compounds, including the complete degradation pathways of the ferulate, benzoate and vanillin degradation (Fig. 3), which are intermediate derivatives of lignin degradation [100, 101] and whose product is acetyl-CoA, a key metabolic compound for biomolecules synthesis and energy generation [102]. Moreover, the presence of extradiol and intradiol dioxygenases related to the ring-cleavage of aromatic compounds have also been found in metagenomic studies performed in non-intestinal bacteria *Acinetobacter* and *Serratia* from *Ips subelongatus* [103]. Furthermore, in vitro assays with the bacteria *Novosphingobium* isolated from galleries of *D. valens* have documented the participation of ring-cleavage dioxygenases in the degradation of the phenolic compound naringenin [25]. Since bark beetles apparently lack ring-cleavage dioxygenases despite the negative effects that phenolic compounds have on insect fitness [104, 105], the presence of these enzymes in bark beetle symbionts could be relevant for increasing insect survival when dealing with phenolic compounds.

### Phase II of detoxification

The genes encoding for xenobiotic degradation enzymes of this phase were integrated by 27 different transferases present in all microorganisms (Supplemental 6). The most abundant are glutathione S-transferase (GST, EC:2.5.1.18) known as conjugating glutathione to xenobiotics, which improves the compounds’ solubility and, consequently, their excretion [106]. Unfortunately, there are few studies on GSTs´ functional role in microbial symbionts and bark beetles, despite being commonly found in metagenomics and transcriptomics studies [80, 107].

The bacterial GSTs found in this work were classified as cytosolic (cGST), being predominantly GstA (B1-1) of the Beta class (Supplemental Table 4). This agrees with those reported for Enterobacteria in which beta GSTs are also the most common class [108]. Moreover, other GST classes such as yfcF and yfcG Nu-like identified in the analyzed bacteria, had also been reported in *Escherichia coli* and associated with mechanisms against oxidative stress generated by cumene hydroperoxide, a toxic organic compound [109]. Also, a GST Nu class of *Novosphingobium aromaticivorans* showed a lyase activity on aromatic intermediates produced during lignin depolymerization [110]. Therefore, we suggested that the GSTs of *D. rhizophagus* gut bacteria could participate in the oxidative resistance of the toxic environment under the bark and probably contribute to the degradation of aromatic compounds of the tree. This proposal is supported by the presence of genes encoding the 3-oxoadipate CoA-transferase [alpha and beta subunit EC:2.8.3.6], acetyl-CoA acyltransferase [EC:2.3.1.16], and 3-oxoadipyl-CoA thiolase [EC:2.3.1.174]. These are conjugation enzymes that participate in the final steps of the degradation of aromatic lignin derivatives (Fig. 3) and they have also been found in fungi phytopathogens and ectosymbionts of beetles involved in the degradation of phenolic compounds [111].

The most abundant yeast GSTs are GTT1, GTT2 and URE2 cytosolic (Supplemental Table 5). These classes have been recognized in *S. cerevisiae* and *Schizosaccharomyces pombe* yeasts as part of the defense systems against environmental and oxidative stress, because they preferably act on reactive oxygen species (ROS) derived cellular metabolism [112–114]. Furthermore, the URE2 class has been found to be associated with the degradation of wood by the fungus *Phanerochaete chrysosporium* [115]. In this regard, the function of GSTs present in *D. rhizophagus* intestinal yeasts could be directed to mediate monoterpene-induced ROS [116] or to conjugate lignin derivatives.

### Phase III of detoxification

The final step, excretion process of soluble toxic metabolites is mediated by multidrug efflux transporters. Our findings showed the presence of 25 diverse MDR transporters associated with the detoxification process (Supplemental Table 6), of which the families ABC and MFS were the most abundant in bacteria and yeasts from the gut of *D. rhizophagus* (Fig. 5). Specifically, multidrug ABCs such as MacB, DrugE1, DrugE2 and ABCB are present in bacteria, while ABCB, ABCC, and PDR-ABCG are in yeasts (Supplemental Table 6). The ABC transporters mediate tolerance to xenobiotics and toxic compounds in fungi, because they transport these substances across membranes [88, 117]. The experimental results carry out in the fungi *G. clavigera*, a pine pathogenic fungus associated with the bark beetle, the pine saprophyte *Ophiostoma piceae*, and the yeast *C. americana* which are present in the *Dendroctonus*´s gut, showed that the PDR-ABCG transporters confer resistance and tolerance to terpenes, because they could excrete either phase I oxidized or conjugated terpenes during phase II [44, 118].

Conversely, the second family of transporters identified is the multidrug MFS associated with DHA1 and DHA2 and identified in bacteria and yeast (Fig. 7). These transporter systems of ubiquitous proteins, which extrude sugars, drugs, xenobiotics, and small solutes in response to chemiosmotic ion gradients, are also involved in the detoxification of plant defenses [119]. These MFS transporters also confer resistance on bacteria and fungi to tolerate multiple antimicrobial agents, decreasing the intracellular concentration of these substances [120]. MFS transporters in *L. qinlingensis* associated with *D. armandi*, and *G. clavigera* from *D. ponderosae*, are overexpressed after symbionts grow in the presence of terpenes and pine phloem extracts [91, 121]. The Aromatic Acid: H + Symporter Family (MFS) and the Hydrophobe/Amphiphile Efflux-1 Family (RND) in bacteria are associated with the transport of aromatic compounds such as 3-(3-hydroxyphenyl) propanoate, 4-hydroxybenzoate, benzoate, 3-phenylpropionic acid, and shikimate (Supplemental Table 4). In environmental isolates of the genera *Acinetobacte*r and *Pseudomonas*, the activity of aromatic transporters has been elucidated in the degradation of aromatic derivatives from lignin [122]. Also, in *Pseudomonas aeruginosa*, it has been demonstrated, using mutant strains with silenced RND transporters, that the bacterium participates in the tolerance of oxygenated-monoterpenes and terpenes [123]. Finally, the MATE family is the least abundant MDR transporter in all microorganisms (Fig. 5), highlighting the NorM and DinF families in bacteria and the ERC1 family in yeast. MATEs in bacteria are associated with the extrusion of a wide range of antibiotics and hydrophobic toxic compounds [124]. The NorM and DinF transporters have been identified in the phytopathogenic bacteria *Erwinia amylovora* and *Ralstonia solanacearum*, respectively. Their expression is associated with the survival and virulence of these bacteria by growing on plant-produced compounds (flavonoids, alkaloids) and resisting epiphytic bacterial antibiotics [125, 126]. The yeast ERC1 family has been reported in *S. cerevisiae* [127] as a transporter of resistance to ethionine, a methionine antagonist compound identified as an odorant precursor in durian plants [128]. Therefore, MATE transporters found in microorganisms could participate both in the detoxification of phytochemicals, and the resistance to the pressure generated by antagonistic microbiota.

## Conclusions

Members of the gut microbiota of *D. rhizophagus* have genes encoding cytochrome P450s, carboxylesterases, laccases, aldehyde and alcohol dehydrogenases, flavin binding-monooxygenases, intradiol and extradiol dioxygenases, glutathione S-transferases, and multidrug resistance transporters involved in various metabolic pathways of xenobiotic detoxification. The bacterial and yeast genes were functionally redundant to some metabolic pathways but mostly complementary for the three phases of detoxification of aromatic and terpene compounds. These results suggest that the microorganisms could play a role in the tolerance and metabolism of host tree defenses during the colonization and life cycle of the insects. This work highlights the importance of bark beetles´ gut microbiota as a supportive strategy in detoxification and requires further research to validate the functionality of the reported genes.

## Supplementary Information

Additional file 1

Supplemental Table 1.

Supplemental Table 2.

Supplemental Table 3.

Supplemental Table 4.

Supplemental Table 5.

Supplemental Table 6.

## Data Availability

The datasets presented in this study can be found in National Center for Biotechnology Information (NCBI) Bioproject database under accession number: PRJNA1269286.
