# Supplementary material for: Comparative genomics of dominant members of the gut core microbiome of the bark beetle, Dendroctonus rhizophagus (Curculionidae: Scolytinae) reveals potential functional complementarity in the detoxification process
Source: BMC Genomics. 2025 Nov 19;26:1064. doi: 10.1186/s12864-025-12279-1 (PMC12628601; doi:10.1186/s12864-025-12279-1)
Supplement: Supplementary file 1 — Additional file 1 [file 12864_2025_12279_MOESM1_ESM.pdf]

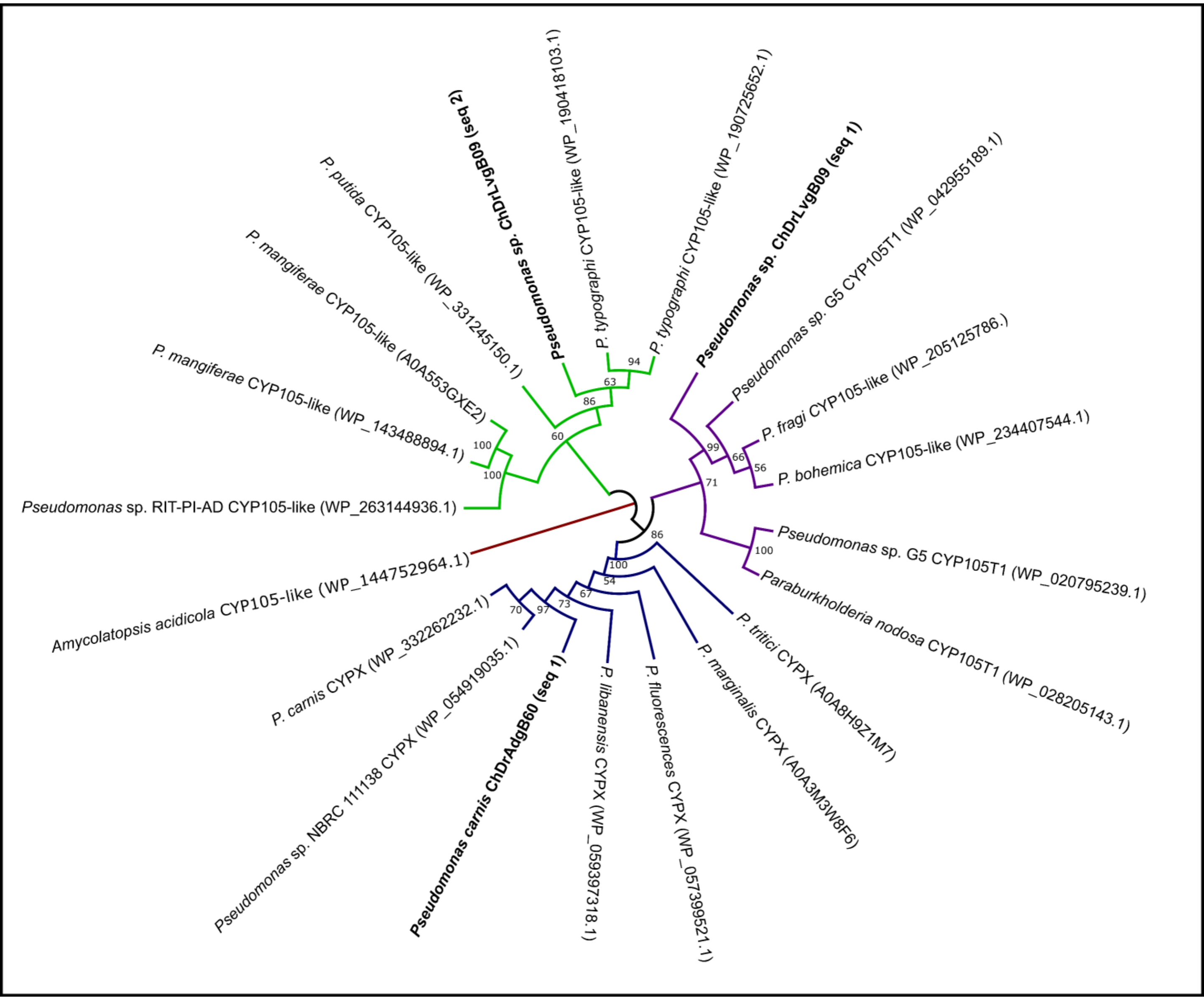

Supplemental Fig. 1. Maximum likelihood phylogeny of *Pseudomonas* spp. CYP450. The tree consisted of CYP450 sequences (429 aa) from 17 related taxa, three CYP450s from *Pseudomonas carnis* ChDrAdgB60 and *Pseuomonas* sp. ChDrLvgB09 highlighted in bold, and CYP105-like root taxa from *Amycolatopsis acidicola*. Their accession numbers are shown within the parentheses and bootstrap values on the nodes. The best amino acid substitution model was LG+G with gamma shape parameter = 1.179. The purple branches correspond to CYP105T1 family, blue branches to CYPX family (Terpene synthase family 2), green branches to CYP105-like, and red branch to the rooted taxa

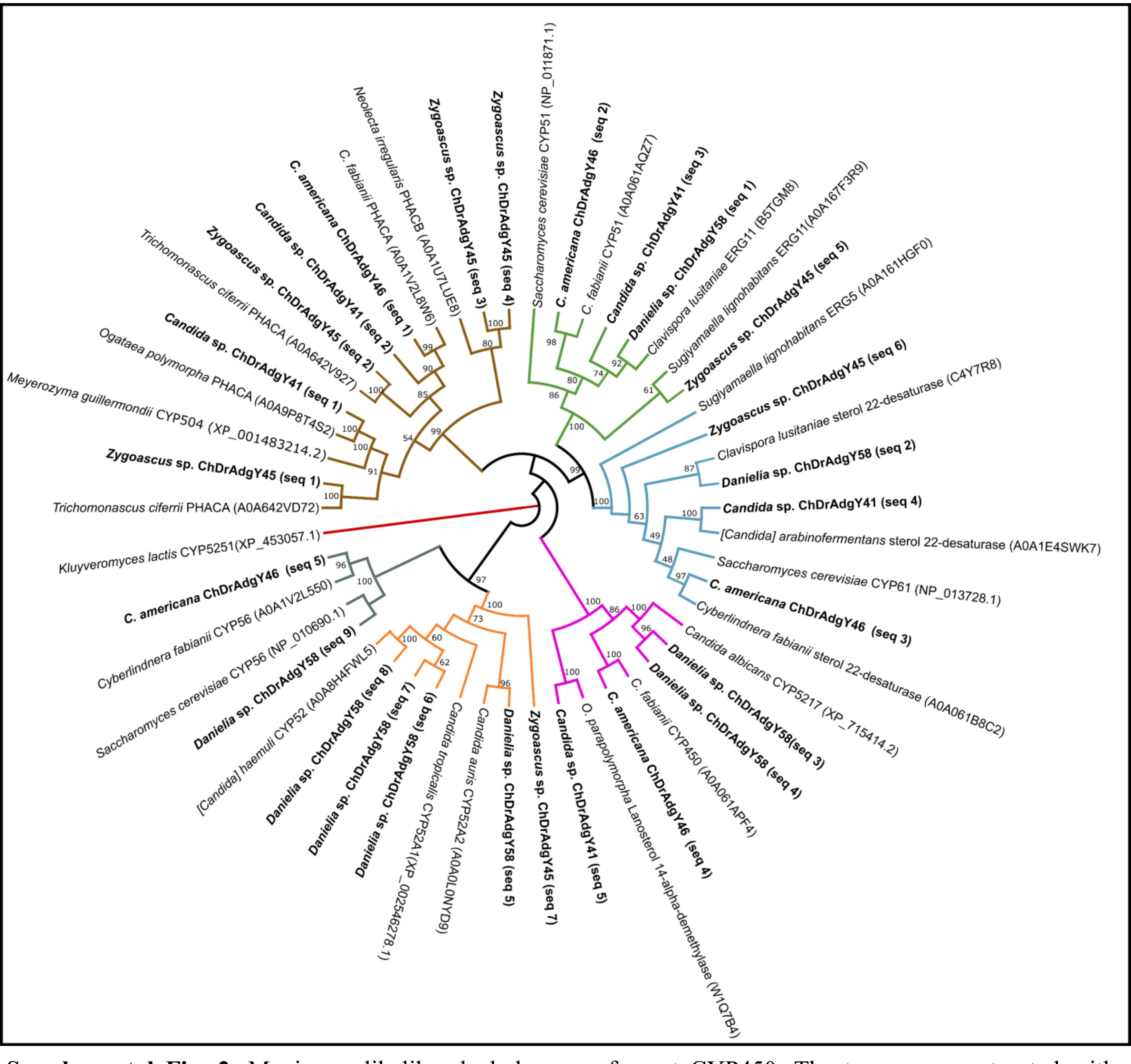

Supplemental Fig. 2. Maximum likelihood phylogeny of yeast CYP450. The tree was constructed with CYP450 sequences (660 aa) from 23 related taxa, 26 yeast CYP450s from gut yeast of *D. rhizophagus* (bold), and one CYP5251 root taxa from *Kluyveromyces lactis*. Their accessions are shown within the parentheses and bootstrap values on the nodes. The best amino acid substitution model was LG+G+I with gamma shape parameter = 2.34 and proportion of invariant = 0.021. The brown branches correspond to CYP504 (PhacA/B), green branches to CYP51 (ERG11), blue light to CYP61 (ERG5), pink branches to CYP5216/CYP52XX, orange branches to CYP52 (ALK), and one red branch to the rooted taxa
